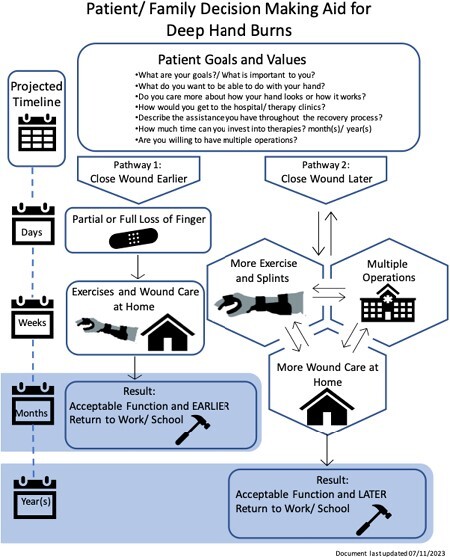# 77 Utility of a Decision-Making Aid for Patient Centered Management of Deep Hand Burns

**DOI:** 10.1093/jbcr/irae036.069

**Published:** 2024-04-17

**Authors:** Kristine A Parker, Jody I Sabel, Caitlin M Orton, Clifford C Sheckter, Tam N Pham

**Affiliations:** University of Washington Regional Burn Center at Harborview, Seattle, WA; UW Medicine Regional Burn Center, Harborview Medical Center, Seattle, WA; Stanford/Santa Clara Valley Medical Center, San Jose, CA; University of Washington, Harborview Burn Centre, Seattle, WA; University of Washington Regional Burn Center at Harborview, Seattle, WA; UW Medicine Regional Burn Center, Harborview Medical Center, Seattle, WA; Stanford/Santa Clara Valley Medical Center, San Jose, CA; University of Washington, Harborview Burn Centre, Seattle, WA; University of Washington Regional Burn Center at Harborview, Seattle, WA; UW Medicine Regional Burn Center, Harborview Medical Center, Seattle, WA; Stanford/Santa Clara Valley Medical Center, San Jose, CA; University of Washington, Harborview Burn Centre, Seattle, WA; University of Washington Regional Burn Center at Harborview, Seattle, WA; UW Medicine Regional Burn Center, Harborview Medical Center, Seattle, WA; Stanford/Santa Clara Valley Medical Center, San Jose, CA; University of Washington, Harborview Burn Centre, Seattle, WA; University of Washington Regional Burn Center at Harborview, Seattle, WA; UW Medicine Regional Burn Center, Harborview Medical Center, Seattle, WA; Stanford/Santa Clara Valley Medical Center, San Jose, CA; University of Washington, Harborview Burn Centre, Seattle, WA

## Abstract

**Introduction:**

Patients with deep finger and hand burns often face the challenge of early amputation vs. tissue salvage and complex rehabilitation. The decision-making aid for deep hand burns was developed to help patients/families identify and understand options and recovery goals. The visual aid outlines distinct pathways with anticipated rehabilitative needs across a timeline of months to years. This project aims to consumer test this tool to ensure it can be presented in an understandable, useful and acceptable manner to patients/families.

**Methods:**

We conducted cognitive interviews with individuals living with deep hand burns and their family members. Interviews addressed the utility of the decision-making aid, burn injury and rehabilitation characteristics, use of the decision-making aid to outline patient goals and values, and the medical pathways, including surgical, rehabilitative, and wound care expectations anticipated with each pathway. Interviews were transcribed and coded by three analysts using both deductive and inductive approaches.

**Results:**

A total of 10 interviews represented diverse perspectives (e.g., burn survivor versus family member/caregiver, time since injury, severity of injury). Within the knowledge, attitudes and beliefs framework, six themes emerged: i) time since injury and severity of injury both impact the ability to interpret recovery, ii) a gap in understanding exists about the recovery process (e.g., outcomes, therapy follow up, more operations, timelines), iii) participants prefer clear and concise messaging regarding options, iv) a knowledgeable advocate on deep hand burns (such as a burn therapist) should be included in discussions on treatment options, v) the decision-making aid needs to be more engaging, vi) repeated use of the tool can support decision-making at different stages of recovery.

**Conclusions:**

The decision-making aid is useful in outlining the patient’s values and recovery goals. It facilitates conversations and helps identify gaps in knowledge. This tool should be utilized multiple times to revisit treatment options throughout the recovery process.

**Applicability of Research to Practice:**

For individuals with complex burn injuries, clinicians should consider the routine use of visual decision-making aids to better understand the patient’s values and recovery goals.